# *Tex46* knockout male mice are sterile secondary to sperm head malformations and failure to penetrate through the zona pellucida

**DOI:** 10.1093/pnasnexus/pgae108

**Published:** 2024-03-12

**Authors:** Yoshitaka Fujihara, Haruhiko Miyata, Ferheen Abbasi, Tamara Larasati, Kaori Nozawa, Zhifeng Yu, Masahito Ikawa, Martin M Matzuk

**Affiliations:** Department of Advanced Medical Technologies, National Cerebral and Cardiovascular Center, Suita, Osaka 564-8565, Japan; Research Institute for Microbial Diseases, Osaka University, Suita, Osaka 565-0871, Japan; Center for Drug Discovery, Baylor College of Medicine, Houston, TX 77030, USA; Department of Pathology & Immunology, Baylor College of Medicine, Houston, TX 77030, USA; Research Institute for Microbial Diseases, Osaka University, Suita, Osaka 565-0871, Japan; Research Institute for Microbial Diseases, Osaka University, Suita, Osaka 565-0871, Japan; School of Medicine, University of California, Davis, Sacramento, CA 95817, USA; Research Institute for Microbial Diseases, Osaka University, Suita, Osaka 565-0871, Japan; Department of Advanced Medical Technologies, National Cerebral and Cardiovascular Center, Suita, Osaka 564-8565, Japan; Center for Drug Discovery, Baylor College of Medicine, Houston, TX 77030, USA; Department of Pathology & Immunology, Baylor College of Medicine, Houston, TX 77030, USA; Center for Drug Discovery, Baylor College of Medicine, Houston, TX 77030, USA; Department of Pathology & Immunology, Baylor College of Medicine, Houston, TX 77030, USA; Research Institute for Microbial Diseases, Osaka University, Suita, Osaka 565-0871, Japan; Center for Infectious Disease Education and Research (CiDER), Osaka University, Suita, Osaka 565-0871, Japan; The Institute of Medical Science, The University of Tokyo, Minato-ku, Tokyo 108-8639, Japan; Center for Drug Discovery, Baylor College of Medicine, Houston, TX 77030, USA; Department of Pathology & Immunology, Baylor College of Medicine, Houston, TX 77030, USA

**Keywords:** CRISPR-Cas9, fertilization, infertility, spermatogenesis, zona pellucida

## Abstract

Each year, infertility affects 15% of couples worldwide, with 50% of cases attributed to men. It is assumed that sperm head shape is important for sperm-zona pellucida (ZP) penetration but research has yet to elucidate why. We generated testis expressed 46 (*Tex46*) knockout mice to investigate the essential roles of TEX46 in mammalian reproduction. We used RT-PCR to demonstrate that *Tex46* was expressed exclusively in the male reproductive tract in mice and humans. We created *Tex46*^−/−^ mice using the Clustered Regularly Interspaced Short Palindromic Repeats (CRISPR)-CRISPR-associated protein 9 (Cas9) system and analyzed their fertility. *Tex46* null spermatozoa underwent further evaluation using computer-assisted sperm analysis, light microscopy, and ultrastructural microscopy. We used immunoblot analysis to elucidate relationships between TEX46 and other acrosome biogenesis-related proteins. Mouse and human TEX46 are testis-enriched and encode a transmembrane protein which is conserved from amphibians to mammals. Loss of the mouse TEX46 protein causes male sterility primarily due to abnormal sperm head formation and secondary effects on sperm motility. *Tex46* null spermatozoa morphologically lack the typical hooked sperm head appearance and fail to penetrate through the ZP. Electron microscopy of the testicular germ cells reveals malformation of the acrosomal cap, with misshapen sperm head tips and the appearance of a gap between the acrosome head and the nucleus. TEX46 is essential for sperm head formation, sperm penetration through the ZP, and male fertility in mice, and is a putative contraceptive target in men.

**Significance Statement**

In our study, we unveil the crucial role of TEX46 in sperm head formation and male fertility. Unlike typical globozoospermia, *Tex46* null spermatozoa primarily exhibit a unique blunted hook-shaped sperm head, resulting in male sterility. This phenotype causes mechanical interaction defects with the oocyte zona pellucida (ZP), thereby impacting ZP penetration, and having secondary effects on sperm motility. TEX46's shifting localization during spermiogenesis contributes to sperm head formation and stability. This discovery not only deepens our understanding of sperm biology but also holds promise for potential applications in infertility treatments and male contraceptives, with implications for human reproduction. Our findings shed light on a conserved mechanism that could have broader relevance across species, warranting future investigations.

## Introduction

A tool specialized for fertilization, the spermatozoon is a highly conserved structure across many species. In mammals, all spermatozoa have the same morphology: a head, consisting of the acrosome and nucleus; a midpiece, composed of hundreds of energy-producing mitochondria; and a tail, an appendage necessary for movement. However, the actual shape of a mammalian spermatozoon may differ between species. For example, human spermatozoa are cone-shaped, while mice have a distinct sickle-like shape. There are many theories as to why spermatozoa have these different shapes. For example, sperm length may be linked to the size of the female reproductive tract (1, 2). However, little is known about why the shape of the sperm head differs between species and if there is an adaptive reason within a species.

Globozoospermia, or round-headed spermatozoa, is a rare phenotype in humans and other mammals that is a known cause of infertility and is linked to improper acrosomal membrane formation during the maturation stage of spermatogenesis (3). Recently, proteins that, when mutated in mice, cause globozoospermia have been discovered, including DPY19L2, FAM209, FAM71F1, FAM71F2, GBA2, GOPC, PDCL2, PICK1, SPACA1, SSMEM1, and ZPBP1 (4–13). Because the acrosome is either lost or misshapen, the acrosome reaction does not occur and the mutant spermatozoa cannot fertilize oocytes. In this case, it is specifically because of the acrosomal insufficiency that globozoospermia and infertility occur. Round-headed spermatozoa are unable to penetrate the zona pellucida (ZP) due to their abnormally shaped sperm heads. Currently, outside of globozoospermia, there is little that shows the relationship between ZP penetration and sperm head.

Another important factor for fertilization is the penetration of the ZP. Mouse SPACA4 (14) and hamster ACROSIN (15) have been found to be important for ZP penetration. Mouse SPACA4 is an inner-acrosomal membrane protein and hamster ACROSIN is a sperm acrosomal enzyme. When these proteins are mutated, the necessary protein–protein interaction between the mutant spermatozoon and an oocyte is disrupted, rendering the male infertile. However, the oocyte interacting partners of these sperm proteins have not yet been determined.

In this paper, we examined testis expressed 46 (TEX46), a protein that shares an integral component of the membrane according to RefSeq. UniProt shows a helical transmembrane domain at positions 34–51. We show that mouse TEX46 is a testicular germ cell-specific protein expressed during the late stages of spermiogenesis. We characterized a coding sequence deletion of mouse *Tex46* generated via the Clustered Regularly Interspaced Short Palindromic Repeats (CRISPR)-CRISPR-associated protein 9 (Cas9) system and suggest that TEX46 is essential for sperm head formation. Proteins are important for acrosome formation and protein–protein interactions during ZP penetration were all normal. However, through analysis of the mutant spermatozoa, we found that the hooked shape of mouse spermatozoa is required for penetration through the ZP.

## Results

### Mouse *Tex46* is a conserved and testis-enriched gene

**Fig. 1.**
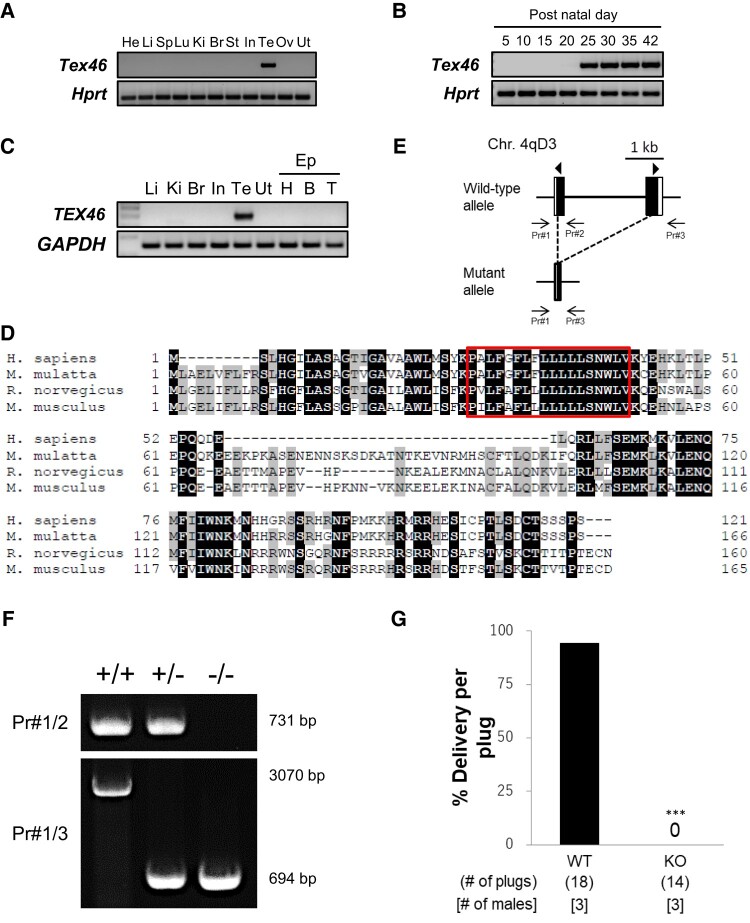
Characterization of TEX46 and generation of *Tex46*^−/−^ mice via the CRISPR-Cas9 system. A) The expression of mouse *Tex46* in various organs was examined by RT-PCR analysis. *Tex46* is testis-enriched. *Hprt* was used as an expression control. He, heart; Li, liver; Sp, spleen; Lu, lung; Ki, kidney; Br, brain; St, stomach; In, intestine; Te, testis; Ov, ovary; Ut, uterus. B) The expression of mouse *Tex46* on indicated postnatal days in the testis was examined by RT-PCR. *Tex46* begins expression at postnatal day 25. *Hprt* was used as an expression control. C) The expression of human *TEX46* in various organs was examined by RT-PCR. *TEX46* is testis-specific. *GAPDH* was used as an expression control. Li, liver; Ki, kidney; Br, brain; In, intestine; Te, testis; Ut, uterus; Ep, epididymis; H, head; B, body; T, tail. D) Sequence similarity of TEX46 proteins in various organisms: Humans, macaques, rats, and mice. Black and gray indicate a match in all four species and three species, respectively. A box indicates a predicted transmembrane region (PxLFxFLxLLLLLSNWLV). E) Schematic of the *Tex46* locus and CRISPR-Cas9 targeting scheme. Arrowheads indicate guide RNAs for Cas9 cleaving. Arrows represent primers. F) Genotyping *Tex46*^−/−^ mice via PCR amplification using primers 1, 2, and 3. G) The average percentage of delivery per plug. *n* = 3 males each for WT and *Tex46*^−/−^ (KO) mated with WT females. ***P < 0.001, Student's t test.

Mouse *Tex46* is a testis-enriched gene located on chromosome 4 that encodes a 165 amino acid protein. RT-PCR analysis in mouse tissues showed strong expression of *Tex46* in the adult testis (Fig. 1A). Expression was detectable at postnatal day 25 and beyond in the testis (Fig. 1B), indicating that the gene is expressed during the haploid stage of spermatogenesis. RT-PCR using human samples showed similar enriched expression in the testis (Fig. 1C). Human and mouse TEX46 share 50% similarity in protein sequence, and humans, rhesus macaque, rats, and mice have a predicted 18 amino acid transmembrane domain (Fig. 1D).

### Generation of *Tex46* knockout mice

To understand the importance of TEX46 in fertility, we used the CRISPR-Cas9 system to first create a mouse that was found to have a five base pairs (bp) deletion (Fig. S1A). Using the same guide RNA (gRNA) targeting exon 1, we created another mouse that had a large portion of the coding region deleted between exon 1 and 2 (Fig. 1E). We obtained a mutant mouse with a 2,376 bp deletion. Homozygous mutant mice (*Tex46*^−/−^) had no overt abnormalities. This large deletion was confirmed via PCR using primers 1 and 2 to detect the wild-type (WT) allele and primers 1 and 3 to detect the 2,376 bp deleted allele (Fig. 1F) and by sequence analysis (Fig. S1B).

### *Tex46* is required for male fertility and sperm head formation

**Fig. 2.**
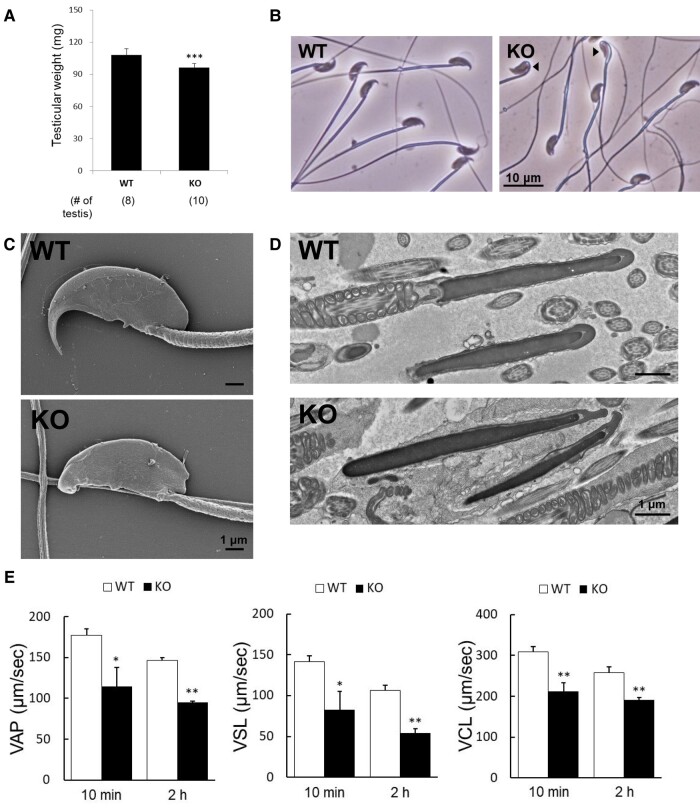
Phenotypic analysis of *Tex46*^−/−^ male mice. A) The average testicular weight. *n* = 4 males for WT and *n* = 5 *Tex46*^−/−^ (KO). ***P < 0.001, Student's t test. B) Phase-contrast microscopy of spermatozoa from the cauda epididymis. Scale bar: 10 μm. There are two populations of *Tex46* null (KO) spermatozoa, blunted tips of the sperm heads that are bent (arrowheads) vs. nonbent. C) SEM observation of mature spermatozoa extracted from the cauda epididymis. *Tex46* null (KO) spermatozoa showed blunted tips of the sperm head. Scale bar: 1 μm. D) TEM observation of mature spermatozoa in the testis. *Tex46* null (KO) sperm heads have a distinct space between the acrosomal cap and nucleus, as well as abnormal blebbing. Scale bar: 1 μm. E) Sperm motility analysis of *Tex46* null (KO) epididymal spermatozoa. Sperm motility results using CASA. VAP, average path velocity; VSL, straight-line velocity; VCL, curvilinear velocity. *n* = 4. *P < 0.05, **P < 0.005, Student's t test.

To test the fertility of the mutant and control male mice, *Tex46*^−/−^ males and WT controls were mated with female mice for 2 months. While the control mice had 18 plugs with an average of 94.4% (17/18 plugs) delivery per plug, the three *Tex46*^−/−^ (knockout: KO) males were sterile despite the formation of 14 copulatory plugs (Fig. 1G, ***P < 0.001). The two lines (5 and 2,376 bp deletions) of KO mice had the exact same infertility phenotype. To investigate why *Tex46*^−/−^ males were sterile, we first compared the testicular weight of WT and mutant mice and found significantly reduced size (WT 107.9 ± 5.8 mg vs. *Tex46*^−/−^ 96.3 ± 3.7 mg) (Fig. 2A, ***P < 0.001). Next, we compared the morphology of cauda epididymal spermatozoa via light microscopy and found two populations of spermatozoa, one that had bent heads and one that had normal heads. However, all sperm heads lacked a distinct hook shape (Fig. 2B). This was confirmed using electron microscopy of cauda epididymal and testicular spermatozoa (Figs. 2C, D and S2A). Scanning electron microscopy (SEM) revealed the abnormal formation of the sperm head hook. In WT mice, the sperm head has a curved, sharp point. However, *Tex46* null spermatozoa have blunted tips. Furthermore, we also observed cauda epididymal spermatozoa with bent heads (Fig. S2A). In WT spermatozoa, the nucleus is an electron–dense structure while the rounded cap is the acrosome. This acrosome tethers to the nucleus to form a sheath around the rostral part of the nucleus. Transmission electron microscopy (TEM) exhibited abnormal dissociation of the acrosome during cap biogenesis (Fig. 2D). In a few spermatozoa, the rostral tip of the acrosome blebs out toward the front. There is a distinct space around the nucleus and the acrosome. While there tends to be minimal space between the rostral most tip of the nucleus and acrosome in WT spermatozoa, many of the mutant sperm have a distinct separation between the two structures. In other spermatozoa, the acrosome cap does not form a tight bond between the structures and blebs out toward the sides. Without *Tex46*, proper acrosome cap formation does not occur, leading to a lack of hook-shaped sperm heads.

### *Tex46* null spermatozoa show motility defects using a computer-assisted sperm analyzer

**Fig. 3.**
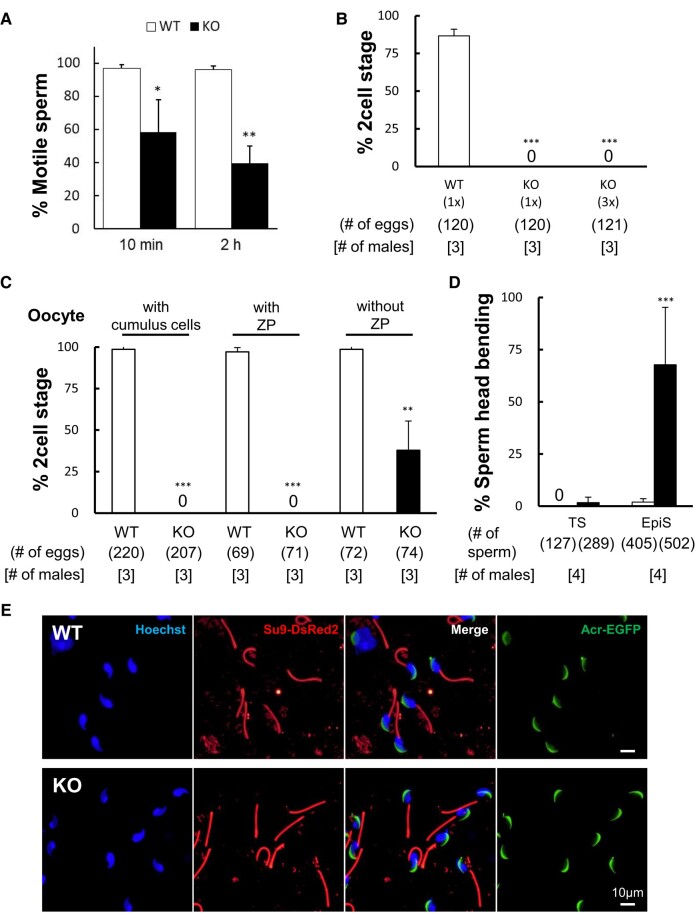
*Tex46* mutant spermatozoa have impaired motility and ZP penetration. A) Percent of motile sperm after capacitation in TYH medium for 10min and 2 h. *Tex46* null (KO) epididymal spermatozoa showed a significantly decreased motility. *P < 0.05, **P < 0.005, Student's t test. B) In vitro fertilization assay of *Tex46*^−/−^ male mice using different concentrations of spermatozoa. *Tex46* null (KO) spermatozoa were unable to fertilize any oocytes, even after concentration was increased from 1 × (2 × 10^5^ sperm/mL) to 3 × (6 × 10^5^ sperm/mL). *n* = 3. ***P < 0.001, Student's t test. C) In vitro fertilization assay of *Tex46*^−/−^ male mice using cumulus-intact, cumulus-free, and ZP-free oocytes. *Tex46* null (KO) spermatozoa were unable to fertilize cumulus-intact (with cumulus cells) and cumulus-free (with ZP) oocytes. In contrast, the percentage of oocytes at the two-cell stage was partially rescued with ZP-free (without ZP) eggs. **P < 0.005, ***P < 0.001, Student's t test. D) Population of bent and nonbent sperm heads in the testis and epididymis. The primary defect in *Tex46*^−/−^ (KO) mice is sperm head formation in the testis. Sperm head-tail junction bending is the secondary effect of *Tex46*^−/−^ mice during the epididymal transition. *n* = 4 males. ***P < 0.001, Student's t test. E) Observation of the acrosome and flagella using EGFP expressed under the *Acrosin* promoter (Acr-EGFP) and mitochondria-targeted DsRed2 in the principal piece (Su9-DsRed2) in *Tex46* null spermatozoa. Although almost all sperm heads of testicular *Tex46* null (KO) spermatozoa are blunted, they nevertheless remain straight like those of WT spermatozoa. Scale bar: 10 μm.

To uncover if the *Tex46* null spermatozoa were infertile due to motility defects, we used a computer-assisted sperm analyzer (CASA) to categorize *Tex46* null mouse sperm motility issues. After incubation in a capacitating medium for 10 and 120 min, the *Tex46* null spermatozoa had decreased average path velocity (VAP) (*Tex46*^−/−^ = 114.8 ± 22.7 μm/s, WT = 177.0 ± 8.1 μm/s at 10 min [*P < 0.05]; *Tex46*^−/−^ = 95.0 ± 2.0 μm/s, WT = 146.5 ± 3.1 μm/s at 120 min [**P < 0.005]), straight-line velocity (VSL) (*Tex46*^−/−^ = 82.7 ± 22.9 μm/s, WT = 141.8 ± 7.1 μm/s at 10 min [*P < 0.05]; *Tex46*^−/−^= 54.5 ± 5.1 μm/s, WT = 106.7 ± 6.2 μm/s at 120 min [**P < 0.005]), and curvilinear velocity (VCL) (*Tex46*^−/−^ = 211.9 ± 21.1 μm/s, WT = 308.3 ± 13.8 μm/s at 10 min [**P < 0.005]; *Tex46*^−/−^ = 191.1 ± 6.6 μm/s, WT = 258.5 ± 14.1 μm/s at 120 min [**P < 0.005]) (Fig. 2E). Overall motility was also reduced at both the 10 min and 2-h mark (*Tex46*^−/−^ = 58.5 ± 19.5%, WT = 96.8 ± 2.5% at 10 min [*P < 0.05]; *Tex46*^−/−^ = 39.8 ± 10.1%, WT = 96.0 ± 2.4% at 120 min [**P < 0.005]) (Fig. 3A). These results suggest that about 50% of *Tex46* null spermatozoa are immotile when analyzed in vitro. However, when we visualized the spermatozoa via TEM, there were no abnormalities in the sperm flagellar cross-sectional structure (Fig. S2B).

### *Tex46* null spermatozoa have defective zona pellucida penetration

Although in vivo fertility is critically impacted in *Tex46*^−/−^ mice, we explored if in vitro fertilization was also affected. After obtaining 361 unfertilized oocytes, we separated them into three conditions and then added 1 × concentration of WT, 1 × concentration of *Tex46* null spermatozoa, and 3 × concentration of *Tex46* null spermatozoa to each dish. We added 3 × concentration of *Tex46* null spermatozoa because ∼40% of spermatozoa were motile after 2 h so we wanted to increase the concentration of motile sperm to match approximately the number of active WT spermatozoa. Even when we added a similar concentration of motile mutant sperm compared to the WT, none of the oocytes in the experimental conditions were fertilized [WT fertilization rate 86.7 ± 4.3%] (Fig. 3B). In this condition, oocytes kept their cumulus cells. To understand where the sperm fertilization defect occurs, we looked at three different oocyte conditions: cumulus-intact (with cumulus cells), cumulus-free ZP-intact (with ZP), and cumulus-free ZP-free (without ZP). *Tex46* null spermatozoa were unable to fertilize cumulus-intact [WT fertilization rate 98.6 ± 2.6%] and cumulus-free ZP-intact [WT fertilization rate 97.1 ± 2.6%] oocytes; however, 37.8 ± 17.6% of *Tex46* null spermatozoa fused with ZP-free oocytes [WT fertilization rate 98.6 ± 2.4%] (Fig. 3C, **P < 0.005 and ***P < 0.001). We found that *Tex46* null spermatozoa can bind to the ZP in vitro, although at a reduced capacity (*Tex46*^−/−^ 40.5 ± 14.6 sperm per oocyte [total 90 oocytes]; WT 108.3 ± 31.4 sperm per oocyte [total 90 oocytes], *n* = 3 males). Moreover, we prepared destabilized ZP oocytes (i.e. reduced disulfide bonds in the ZP proteins) by treatment with Center for Animal Resources and Development (CARD) medium (Kyudo Company, Saga, Japan) to enhance sperm penetration through the ZP in vitro; however, nearly all of the *Tex46* null spermatozoa failed to penetrate through the ZP unlike WT spermatozoa (*Tex46*^−/−^ fertilization rate 0.9 ± 0.8% [total 325 oocytes]; WT fertilization rate 89.7 ± 2.2% [total 329 oocytes], *n* = 3 males). This indicates that *Tex46* null spermatozoa cannot penetrate the ZP, but were able to fuse with oocytes in the absence of TEX46.

### *Tex46* null testicular spermatozoa do not display bent sperm heads

As discussed previously, we found two different populations of spermatozoa: bent vs. nonbent. At the sperm head-tail junction, there was a distinct bend in the heads toward their flagella. We wanted to understand at what stage this bending occurred, and, more specifically, how TEX46 plays a role in this. We took spermatozoa from both the testis and cauda epididymis of *Tex46*^−/−^ mice and found that the testicular sperm had <2% of bent heads [*Tex46*^−/−^ 1.7 ± 2.6%; WT 0%] (Fig. 3D). In our TEM data as well, we found that testicular spermatozoa did not have bent heads (Fig. 2D). In contrast, the cauda epididymal sperm had about 70% bending [WT 2.0 ± 1.7% vs. *Tex46*^−/−^ 67.7 ± 27.5%, ***P < 0.001]. Because almost 100% of testicular spermatozoa were not bent, the bending seems to be a secondary effect that occurs during the epididymal sperm transition. Because of the bending, we wanted to see if the structure of the flagella was changed in any way. To examine the integrity of the flagellar mitochondria of spermatozoa, transgenic mice expressing mitochondria-targeted DsRed2 (Su9-DsRed2) were created and analyzed using fluorescent microscopy (Fig. 3E). Both WT and mutant spermatozoa had normal flagellar structures. This was also confirmed via TEM analysis, as no mutations were seen in the 9 + 2 microtubules and surrounding mitochondrial structures (Fig. S2B). The acrosome reaction, a necessary step for mammalian fertilization (16, 17), was another parameter we measured in *Tex46* null spermatozoa. Using the transgenic mice (Fig. 3E), we compared the signalizing of Acr-enhanced green fluorescent protein (EGFP) between WT and KO spermatozoa. There were no issues with the acrosome reaction in mutant mice during 4 h sperm incubation (WT 29.0 ± 13.7%, *Tex46*^−/−^ 43.1 ± 17.7%, *n* = 3 males).

### Relationship between TEX46 and other globozoospermia-related proteins

**Fig. 4.**
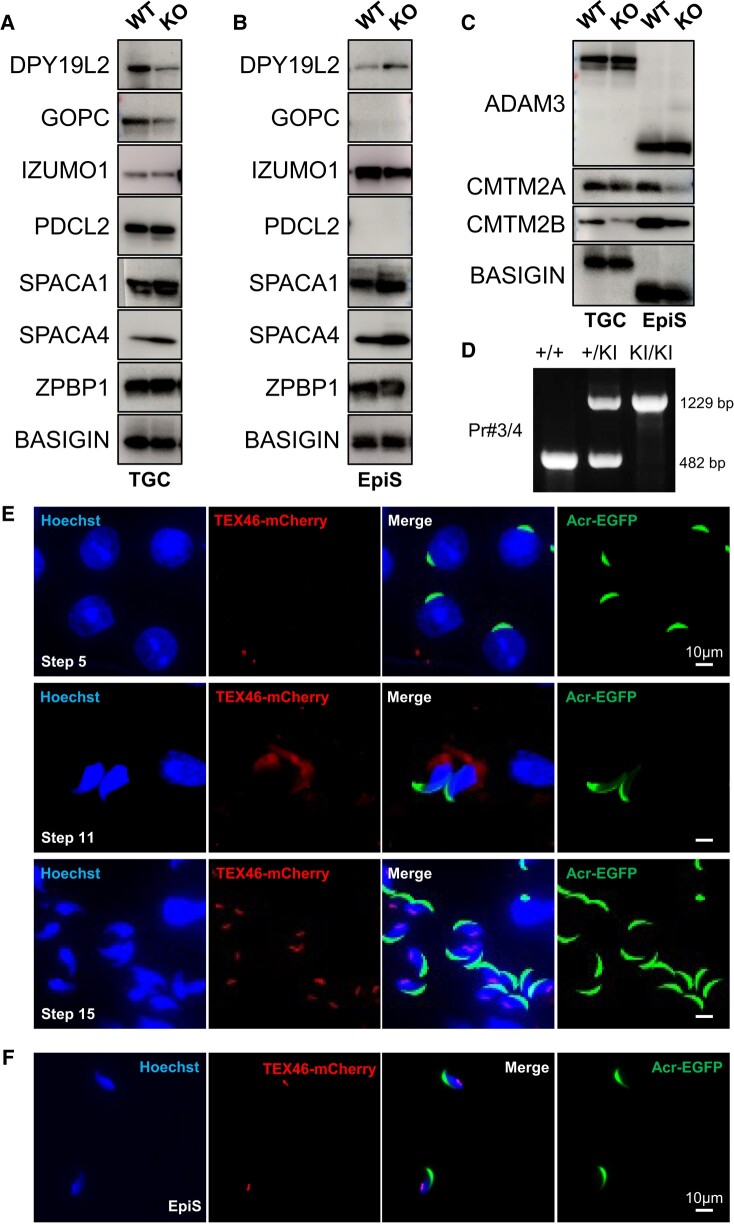
Relationship between globozoospermia-related proteins and localization of TEX46 in spermatozoa. A and B) Immunoblot analysis of proteins related to acrosome biogenesis and ZP penetration in WT and *Tex46* null (KO) TGC (A) and cauda EpiS (B): DPY19L2, GOPC, IZUMO1, PDCL2, SPACA1, SPACA4, and ZPBP1. GOPC and PDCL2 are TGC-specific proteins. BASIGIN was used as a loading control. C) Immunoblot analysis of proteins related to ZP binding and sperm migration into the oviduct in WT and *Tex46* null (KO) TGC and cauda EpiS. ADAM3, CMTM2A, and CMTM2B are sperm membrane proteins that regulate ZP binding and sperm migration through the oviduct. BASIGIN was used as a loading control. D) Genotyping *Tex46-mCherry* knockin (KI) mice via PCR amplification. A mCherry coding sequence was inserted before the terminal codon of the second exon of *Tex46*. E) Immunofluorescence observation of TGC in *Tex46-mCherry* KI mice. In step 5 of spermiogenesis, TEX46-mCherry signals are not seen in round spermatids. mCherry signals are observed around the manchette of the sperm heads in step 11 of spermiogenesis when spermatids are elongating. Then, TEX46 localizes to the postacrosomal region of the sperm head in step 15 of spermiogenesis when mature spermatozoa are formed. Scale bar: 10 μm. F) Immunofluorescence observation of epididymal spermatozoa in *Tex46-mCherry* KI mice. Scale bar: 10 μm.

There are many genes that have been implicated in globozoospermia, including DPY19L2, GOPC, PDCL2, SPACA1, and ZPBP1 (5–7, 10, 12). We conducted immunoblot analysis of WT and *Tex46*^−/−^ testicular germ cells (TGCs) and epididymal spermatozoa (EpiS). The amounts of all of these proteins were not changed between WT and *Tex46* null spermatozoa (Fig. 4A and B). Whereas sperm acrosomal membrane protein IZUMO1 in other mice that display globozoospermia is depleted (5), IZUMO1 levels are normal in this *Tex46* null spermatozoa. Therefore, the phenotype in the *Tex46* null spermatozoa is not related to globozoospermia. Alternatively, SPACA4 is another sperm membrane protein that is important for ZP penetration (14). In *Spaca4* mutant mice, the spermatozoa had no overt morphological abnormalities, but could not penetrate the ZP. However, SPACA4 levels were also normal in *Tex46*^−/−^ mice (Fig. 4A and B). Even though TEX46 was no longer functioning, SPACA4 remained present in the acrosomal membrane, suggesting that the spermatozoa should be able to penetrate the ZP. While it is well understood that the bent sperm cannot penetrate the ZP due to their shape, nonbent sperm should still have SPACA4 on the sperm membrane; thus, in theory, there should be no physiological defects during the ZP penetration stage of fertilization. In addition, sperm membrane proteins ADAM3 and CMTM2A/B remained in *Tex46* null spermatozoa which regulate sperm migration from the uterus into the oviduct (Fig. 4C). These results imply that *Tex46* null spermatozoa can bind to the ZP in vitro (shown in Results). However, as previously stated, despite an increase in concentration of the spermatozoa and an insemination of destabilized ZP oocytes, none of the spermatozoa could fertilize oocytes. This suggests that the hook shape of spermatozoa is a requisite for ZP penetration in mice.

### TEX46 localization pattern in spermiogenesis

We next focused on understanding the localization pattern of TEX46 during spermiogenesis. Unfortunately, we could not obtain TEX46 antibodies. Instead, we created CRISPR-Cas9-mediated knockin (KI) mice expressing mCherry in the C terminus of TEX46 to confirm the localization pattern of the protein (Fig. 4D–F). In the testis from fertile homozygous KI mice during the round spermatid stage of spermatogenesis, there is little-to-no signal throughout the cell (Fig. 4E, step 5). As spermiogenesis occurs, the TEX46 signals are strongly expressed near the manchette of the sperm head (Fig. 4E, step 11). Finally, the protein localizes to the base of the sperm head (Fig. 4E, step 15). In cauda epididymal spermatozoa, TEX46 fluoresces inferior to the acrosomal region, near the sperm head-flagellar junction (Fig. 4F). This suggests that as the spermatozoa mature, TEX46 begins to concentrate at this junction. Without proper TEX46 functions, the sperm head becomes blunted tips in the testis and bends during the epididymal transition.

## Discussion

There are many factors that render male infertility, most often related to sperm motility, sperm ZP binding, and sperm migration into the oviduct (18). Through the creation of CRISPR-Cas9 mutant mice, we reveal that TEX46 is critical for the correct formation of the sperm head and male fertility. We observed a blunted hook-shaped head in *Tex46*^−/−^ mice via electron microscopy (Figs. 2C, D and S2A). This blunted hook shape (i.e. a structural defect) is apparently why penetration through the ZP is halted. However, even without the ZP, the mutant spermatozoa have reduced fertilization ability. CASA exhibits a significant reduction in sperm motility, especially after capacitation for 2 h. However, when we compare this motility data to WT C57BL/6 (B6) inbred mice, we find that the CASA score is similar (2 h incubation: VAP [B6 112 ± 14.7 μm/s vs. KO 95.0 ± 2.0 μm/s], VSL [B6 69.9 ± 11.5 μm/s vs. KO 54.5 ± 5.1 μm/s], VCL [B6 203 ± 14.2 μm/s vs. KO 191 ± 6.6 μm/s]) (19). Sperm head malformations may lead to reduced sperm motility in *Tex46*^−/−^ mice. However, the major cause of infertility in *Tex46*^−/−^ mice is not due to impaired sperm motility.

Typical globozoospermia phenotypes tend to have a complete rounding of the sperm head (3). However, *Tex46* null spermatozoa did not have full globozoospermia. Rather, only the hook was blunted. We hypothesize that TEX46 is necessary for the hook shape, which is implicated in its ability to penetrate the ZP. Furthermore, according to our western blot data, there does not appear to be a relationship between TEX46 and other globozoospermia-related proteins, such as DPY19L2, GOPC, PDCL2, SPACA1, and ZPBP1 (5–7, 10, 12) (Fig. 4A and B). In these globozoospermia-causing proteins, acrosomal membrane protein IZUMO1 expression is also affected. However, in *Tex46* mutant mice, IZUMO1 is expressed normally. Moreover, sperm membrane proteins ADAM3 and CMTM2A/B (18, 20) remain in *Tex46* null spermatozoa, regulating the sperm migrating ability (Fig. 4C). Thus, *Tex46* is neither a globozoospermia-related nor a sperm migration regulating gene.

We previously discovered that sperm glycosylphosphatidylinositol (GPI)-anchored protein SPACA4 is necessary for ZP penetration (14). Although SPACA4 mutant mice have normal sperm head shape and normal motility, the mutant spermatozoa cannot penetrate the ZP. However, even though SPACA4 levels are unchanged in TEX46 mutant mice (Fig. 4A and B), these spermatozoa cannot fertilize oocytes (Fig. 3B and C). Therefore, we hypothesize that the defect in TEX46 is not related to a protein–protein interaction between the spermatozoa and oocyte, but rather another type of interaction, such as a sperm mechanical interaction with the ZP.

About 70% of *Tex46* null cauda epididymal spermatozoa were immotile and bent at the head-tail junction. Obviously, spermatozoa cannot fertilize oocytes if they have bent heads. However, because 30% of them were not bent, we conducted an in vitro fertilization (IVF) experiment to test the fertilizing ability of those spermatozoa. To mimic the conditions of WT spermatozoa, we added three times the concentration of *Tex46* null cauda epididymal spermatozoa, producing a similar number of nonbent spermatozoa. However, when this concentration was added to oocytes, we found that the *Tex46* null spermatozoa still could not fertilize the oocytes (Fig. 3B). In addition, *Tex46* null spermatozoa are unable to penetrate through the ZP that was chemically destabilized although they can bind to the ZP in vitro (Fig. 3C and Results). The nonbent spermatozoa also cannot penetrate the ZP. Therefore, the main cause of infertility in these mice must be due to the lack of sperm head hook. In addition, we found that in the testis, more than 98% of spermatozoa were not bent, similar to WT mice (Fig. 3D and E). However, when they reached the epididymis, almost 70% were bent. This reveals that the bending is a secondary effect of *Tex46* null spermatozoa. This demonstrates that the sperm head hook is important for ZP penetration in mice.

We recently reported that FAM71F1 is important for acrosome formation (8). Unfortunately, we could not obtain an antibody for this protein nor TEX46, and thus cannot see the localization and interaction between these proteins and other globozoospermia or acrosome-related genes. While we may not be able to do further studies due to the lack of antibodies, we did create *Tex46-mCherry* KI mice to observe its localization (Fig. 4D). In the early round spermatid stage, we saw little-to-no TEX46 signals throughout the cell (Fig. 4E). This correlates to postnatal day 25, just as the mRNA is being expressed. It takes some time for the mRNA to convert to protein. However, the protein is still being expressed and is broadly dispersed throughout the spermatid head. As spermiogenesis continues and the sperm head is being formed, TEX46-mCherry signals are detected at the manchette. In the end, the TEX46-mCherry signal is localized to the base of the sperm head. We confirmed this localization pattern in mature epididymal spermatozoa (Fig. 4F). As spermiogenesis occurs, TEX46 functions to form the sperm head as showcased by mCherry observation and knockout analysis. This transition from widely dispersed to localized represents how the protein is first needed to create the proper hook shape, and then later helps to stabilize the sperm head-tail junction. However, there is a possibility that the localization of mCherry-tagged TEX46 has been modified given the addition of mCherry. For further investigation, an antibody is needed to analyze the actual localization of TEX46.

In summary, we report that TEX46 is important for proper sperm head formation, ZP penetration, and male fertility in mice. Identification of TEX46 as a key protein in sperm head formation, especially the signature hook shape of mouse spermatozoa, will help us understand more about the importance of ZP penetration and species differences in sperm head shape. While sperm head shape is assumed to be important for fertilization, we discovered that proper form is necessary for the mechanical penetration of the ZP in mice, and in the *Tex46* knockout case has secondary effects on sperm motility. Future analysis is necessary to determine whether the *Tex46* gene, which is conserved from amphibians to mammals, has a common mechanism for fertilization in each species. Furthermore, human TEX46 exhibits testis-enriched expression, and its function may be conserved in humans as well. Our findings support a potential role of human TEX46 in sperm function and could be used to develop treatments for infertility (in the case of individuals with mutations in human *TEX46*) as well as male-specific contraceptives if molecules could disrupt TEX46 function in vivo.

## Methods

### Animals

All animal experiments were approved by the Animal Care and Use Committees of the National Cerebral and Cardiovascular Center Research Institute and the Research Institute for Microbial Diseases, Osaka University. Human tissue was collected as a part of nonhuman subject research by the Human Tissue Acquisition & Pathology Core at Baylor College of Medicine under the institutional review board-approved Protocol H-14435. Mice were maintained under a 12-h light/dark cycle. In this study, we used a transgenic mouse line (B6D2-Tg[cytomegalovirus {CMV} enhancer fused to the chicken beta-actin {CAG}/Su9-DsRed2, Acr3-EGFP]RBGS002Osb) (21) and generated a genetically modified mouse line, *Tex46* mutant (deleted and mCherry knockin) mice (B6D2-*Tex46* < em1Osb>, B6D2-*Tex46* < em1Ncvc>, and B6D2-*Tex46* < em2(mCherry)Ncvc>). This line will be deposited to the RIKEN BioResource Research Center (http://mus.brc.riken.jp/en/) and the CARD, Kumamoto University (http://card.medic.kumamoto-u.ac.jp/card/english/).

### RT-PCR analysis

RT-PCR analysis was performed as described previously (5). The primers used were: 5′-CAAGAACACAATTTGGCTCCC-3′ and 5′-CCCATCTCAGTCACATTCCG-3′ for mouse *Tex46*; 5′-TGGATATGCCCTTGACTATAATGAG-3′ and 5′-TGGCAACATCAACAGGACTC-3′ for mouse *Hprt*; 5′-CAGAAAACACGGATTGGCAG-3′ and 5′-TTCACTATGCTCTGGTTGCTC-3′ for human *TEX46*; 5′-AATCCCATCACCATCTTCCAG-3′ and 5′-ATGACCCTTTTGGCTCCC-3′ for human glyceraldehyde 3-phosphate dehydrogenase (*GAPDH)*.

### Antibodies

Polyclonal antibodies against mouse CMTM2A, CMTM2B, SPACA1, and ZPBP1 were described previously (6, 7, 20). IZUMO1 and SPACA4 monoclonal antibodies were described previously (22, 23). Other antibodies were purchased from Atlas Antibodies (HPA071264 for DPY19L2 and HPA024018 for GOPC), Proteintech (17407-1-AP for PDCL2), and Santa Cruz Biotechnology (sc-46700 for BASIGIN and sc-365288 for ADAM3). Immunoblot analysis dilutions were 1:500 to 1:1,000.

### Immunoblot analysis

Immunoblot analysis was performed as described previously (5, 24).

### Generation of *Tex46* knockout and *Tex46-mCherry* knockin mice with CRISPR-Cas9

Production of CRISPR-Cas9-mediated mutant mice was performed as described previously (5, 25). The gRNAs and primers used were: 5′-AAAGCTTATCAACCAAGCCA-3′ for the first exon of *Tex46*, 5′-ATGTACTACCGTCACCCCCA-3′ for the second exon of *Tex46*, and 5′-CCATCTCAGTCACATTCCGT-3′ for *Tex46-mCherry* knockin. 1,056 bp single-strand oligonucleotide encoded mCherry was coelectroporated with the gRNA.

The genotyping primers used were: 5′-GAGAGCAGGTGACCTATAGCATCAACTG-3′ for Pr.1, 5′-GCGTGGCTGAAACAGGATGGAACAC-3′ for Pr.2, 5′-CCCTTCAAAGAGTCACTGCTAGCATCACAC-3′ for Pr.3, and 5′-GAGGAGGCAGAAACAACAACAGCTCC-3′ for Pr.4.

### Male fertility test

After sexually mature WT and *Tex46*^−/−^ male mice were caged with 2-month-old B6D2F1 or *Tex46*^−/−^ females for several months, the number of plugs and deliveries in each cage was counted. The average percentage of delivery (delivery per plug) is the total deliveries divided by the number of plugs for each genotype.

### TGCs and sperm morphology

Immunostaining analysis was performed as previously described (5, 26).

### Electron microscope observation

Electron microscope observation was performed as described previously (5).

### Sperm motility analysis

Sperm motility analysis was performed as previously described (5, 19).

### In vitro fertilization

In vitro fertilization using mouse spermatozoa was performed as described previously (27). To prepare cumulus- and ZP-free oocytes, cumulus-oocyte complexes were treated with 0.3 mg/mL hyaluronidase (H4272, Sigma-Aldrich, MO, USA). Then, for ZP removal, the cumulus-free oocytes were treated with 1 mg/mL collagenase (C1639, Sigma-Aldrich, MO, USA) as described previously (28). Two hours of preincubated spermatozoa were inseminated with the differently treated oocytes using Toyoda-Yokoyama-Hoshi (TYH) or Human Tubal Fluid (HTF) mediums for in vitro fertilization. To analyze sperm ZP-binding ability, cumulus-free oocytes were incubated with capacitated spermatozoa in HTF medium (MR-070, Sigma-Aldrich) for 30 min before observation. The bound spermatozoa were fixed with 0.25% glutaraldehyde (Nacalai Tesque, Kyoto, Japan) and were counted. To prepare destabilized ZP oocytes and assess sperm ZP penetration ability, CARD medium and FERTIUP were used under manufacturing protocols (Kyudo Company, Saga, Japan).

### Statistical analysis

Statistical analyses were performed using Student's t test inserted into Microsoft Excel after the data were tested for normality of distribution. Differences were considered significant at *P < 0.05, **P < 0.005, and ***P < 0.001.

## Supplementary Material

pgae108_Supplementary_Data

## Data Availability

All data are available in the article.
