# Supplementary material for: Tex46 knockout male mice are sterile secondary to sperm head malformations and failure to penetrate through the zona pellucida
Source: PNAS Nexus. 2024 Mar 12;3(3):pgae108. doi: 10.1093/pnasnexus/pgae108 (PMC10957234; doi:10.1093/pnasnexus/pgae108)
Supplement: pgae108_Supplementary_Data [file pgae108_supplementary_data.zip › PNASNEXUS-PNASNEXUS-2023-01116RR-s02.pdf]

Figure S2. (Fujihara et al.)

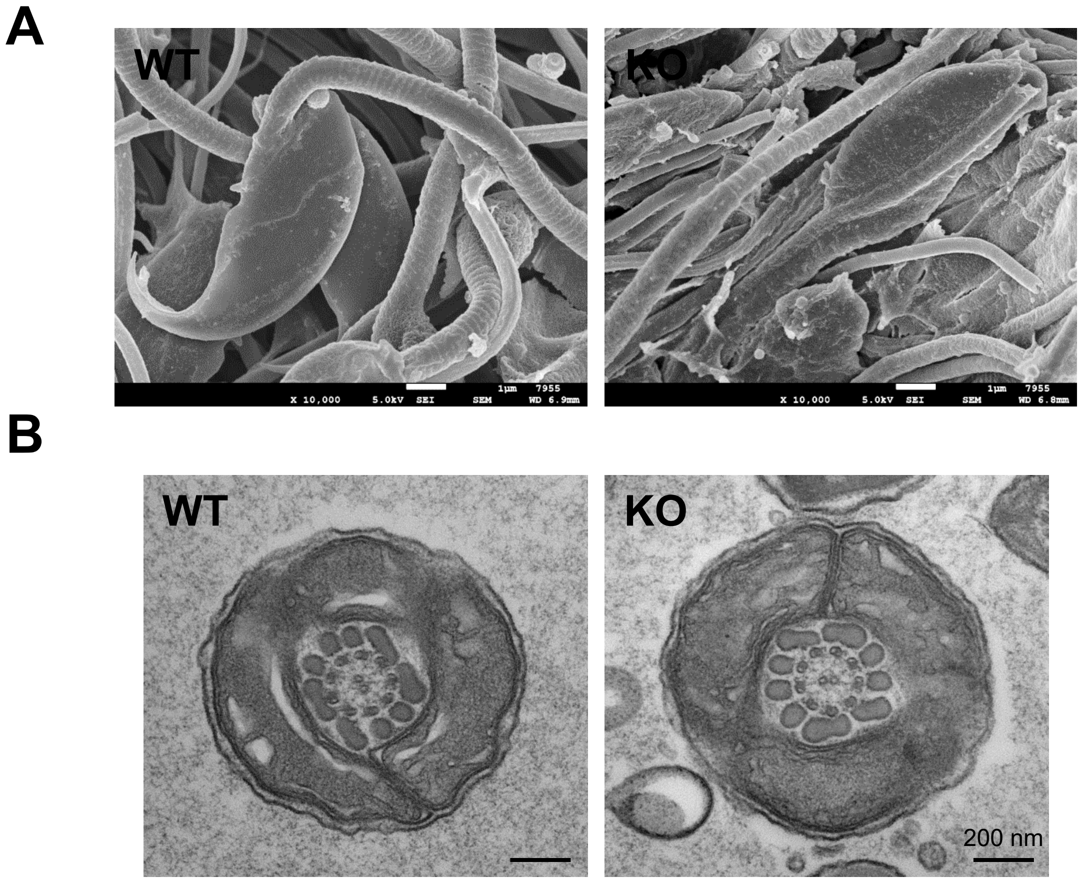

Figure S2. Microscopic observation of *Tex46* mutant spermatozoa
(A) Scanning electron microscopic (SEM) observation of *Tex46* null epididymal spermatozoa. *Tex46*$^{-/-}$ mice bend near the head-tail junction during the epididymal transition. Scale bars: 1 μm.
(B) Transmission electron microscopic (TEM) observation of tail structure in *Tex46* null epididymal spermatozoa. There are no differences between wild-type and *Tex46*$^{-/-}$ mice. Scale bars: 200 nm.
