# Supplementary material for: Tex46 knockout male mice are sterile secondary to sperm head malformations and failure to penetrate through the zona pellucida
Source: PNAS Nexus. 2024 Mar 12;3(3):pgae108. doi: 10.1093/pnasnexus/pgae108 (PMC10957234; doi:10.1093/pnasnexus/pgae108)
Supplement: pgae108_Supplementary_Data [file pgae108_supplementary_data.zip › PNASNEXUS-PNASNEXUS-2023-01116RR-s01.pdf]

Figure S1. (Fujihara et al.)

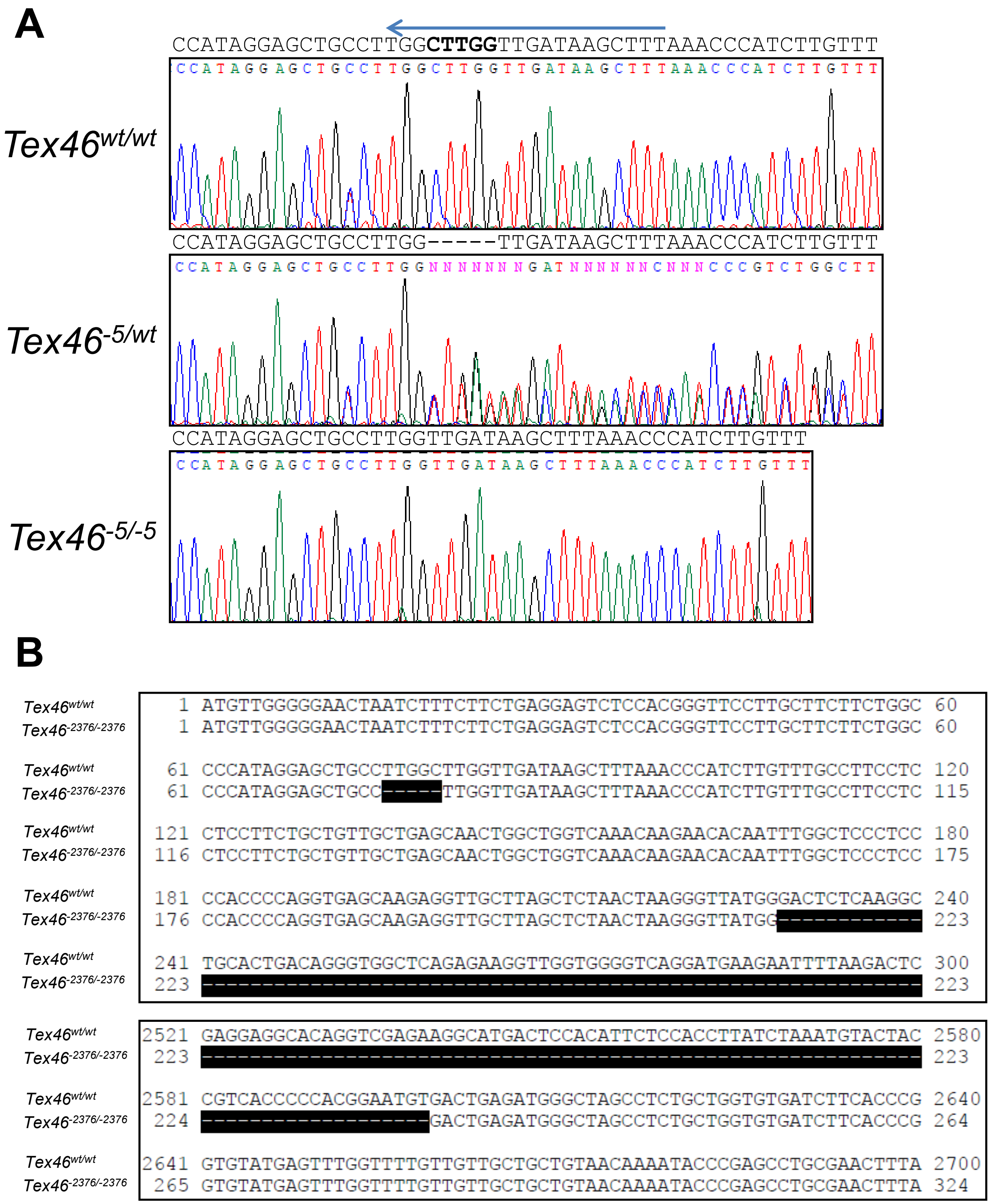

Figure S1. Detailed sequence information of the *Tex46* mutant alleles

(A) Wave pattern sequence of the *Tex46*$^{-5/-5}$ mutant allele (5 bp deletion). The deletion (5'-CTTGG-3') of the first exon causes a frameshift mutation. The blue arrow indicates the guide RNA sequence for Cas9 targeting.

(B) The aligned sequence of the wild-type and *Tex46*$^{-2376/-2376}$ mutant mice (2376 bp deletion). *Tex46*$^{-2376/-2376}$ mutant mice have two deleted regions, within the first exon (5'-TTGGC-3' from 153 bp coding exon) and spanning from the first intron (2068 bp deletion/2107 bp) to the second exon (303 bp deletion/309 bp coding exon). The upper and lower sequences indicate wild-type and mutant genomic DNA sequences, respectively. Black indicates a deleted region in *Tex46*$^{-2376/-2376}$ mutant mice.
